# Smartphone Application–Based Voice and Speech Training Program for Parkinson Disease: Feasibility and Satisfaction Study With a Preliminary Rater-Blinded Single-Arm Pretest and Posttest Design

**DOI:** 10.2196/63166

**Published:** 2025-02-13

**Authors:** Sol-Hee Lee, Jiae Kim, Han-Joon Kim

**Affiliations:** 1 Department of Neurology Seoul National University Hospital Seoul National University College of Medicine Seoul Republic of Korea

**Keywords:** Parkinson disease, speech therapy, mHealth, home-based training, self-delivered, digital health care, app, feasibility, voice therapy, mobile phone, satisfaction, effectiveness, smartphone, apps, single-arm study, mobility, mobile health, acoustic analysis, self-training

## Abstract

**Background:**

Up to 75% of patients with Parkinson disease (PD) experience voice and speech impairments, such as breathy phonation and low speech volume, which worsen over time and negatively impact the quality of life. However, given their increasingly limited mobility, face-to-face speech therapy is often inaccessible. Mobile health (mHealth) apps offer accessible and cost-effective alternatives; yet, their application in PD-specific, self-delivered voice therapy remains underexplored.

**Objective:**

This study aimed to evaluate the feasibility, adherence, and satisfaction of a self-delivered smartphone app for voice therapy in patients with PD, designed to minimize speech-language pathologist involvement while promoting patient independence. In addition, it seeks to assess the preliminary therapeutic effectiveness of the app in addressing voice and speech problems in this population.

**Methods:**

A single-arm, rater-blinded, and pretest and posttest study was conducted between September to November 2023. Patients with PD with voice and speech problems who have no problem with using Android (Google) smartphones were recruited. Participants downloaded the researcher-developed mHealth app on their smartphone and participated in a patient-tailored 5-week home-based speech training program. Each session included 5 stages: breathing, oral motor exercises, loudness, prosody, and functional speaking. The training program consisted of 20 sessions, with participants completing 1 session per day, 4 days per week. Each session lasted approximately 20-30 minutes. Adherence was monitored through app logs, satisfaction was assessed through a phone survey, and therapeutic effectiveness was evaluated using acoustic analysis and auditory-perceptual assessments.

**Results:**

Out of 30 patients were initially recruited, but 2 of them withdrew. Out of 25 participants completed all the training sessions while 3 dropped out. The adherence was above 90% in 20 participants (80%, 20/25), 70% to 90% in 4 (16%, 4/25), and below 70% in 4 (16%, 4/25). Satisfaction was 75% (18/24) among the 24 people who participated in the survey. Significant improvements were observed in all acoustic measures: the maximum phonation time increased from 11.15 (SD 5.38) seconds to 14.01 (SD 5.64) seconds (*P*=.003), and vocal intensity increased from 71.59 (SD 4.39) dB to 73.81 (SD 3.48) dB (*P*<.001) across both sustained phonation and reading tasks. Voice quality scores on the GRBAS (grade, roughness, breathiness, asthenia, and strain) scale improved significantly (all components *P*<.001). Furthermore, 58.3% (14/24) of participants reported subjective improvements in their voice.

**Conclusions:**

This study demonstrates that home-based, self-training speech therapy delivered through a mHealth app is a feasible solution for patients with PD, suggesting that mHealth apps can serve as a convenient and effective alternative to face-to-face therapy by enhancing accessibility and empowering patients to actively manage their condition.

## Introduction

During the progression of patients with Parkinson disease (PD), around 75% of patients experience difficulties with their voice and speech functions at some stage [1]. The main characteristics of speech include breathy phonation, hoarseness, low speech volume, inaccurate articulation, and monotonous speech. These symptoms worsen over time. Consequently, these symptoms can significantly impact the quality of life, affecting physical, emotional, functional, and social well-being [2]. They also hinder communication, daily activities, and social participation. Therefore, rehabilitation is essential for individuals with PD, with speech therapy being particularly important as it helps patients maintain and improve their communication abilities in everyday life [3]. Traditionally, speech therapy involves in-person sessions with a speech-language pathologist (SLP). However, the global COVID-19 pandemic disrupted traditional service delivery models, leading to increased interest in remote rehabilitation and mobile health care technologies as viable alternatives. In addition, the rising demand for rehabilitation due to an aging population, coupled with limited health care resources, further highlights the need to adopt and expand information and communication technology [4].

A review of previous studies has confirmed the feasibility of remote speech-language therapy (SLT) across various disability groups. According to Weidner and Lowman [5], remote SLT is primarily conducted in real-time using commercial software such as Skype (Skype Technologies) and Zoom (Zoom Video Communications), or through custom-developed software installed on computers or tablets. While most remote SLT initiatives have focused on patients with chronic aphasia, some have targeted individuals with PD. Many existing studies have concentrated on delivering the Lee Silverman Voice Treatment LOUD or its extended versions, which are proven to be effective for addressing speech and voice issues in patients with PD, through remote platforms [6-9]. Research on remote SLT for patients with PD has shown that it is just as effective as in-person therapy, with no significant reduction in efficacy [6-10]. Meanwhile, mobile health (mHealth) apps, developed to support users in managing their health independently, have advanced considerably. According to the World Health Organization, mHealth is a broad term encompassing activities that involve the use of smartphones, sensors, personal digital assistants, wireless monitoring devices, or other wireless technologies to deliver public health and health care services [11]. These technologies hold promises for promoting autonomous patient participation in treatment, thus maintaining continuity of care while offering benefits in terms of accessibility and cost [12,13].

To date, most mHealth have been designed and studied with a focus on health outcomes such as nutrition and physical activity, mental health management, and diabetes care, primarily targeting populations with chronic conditions, mental health issues, or obesity [14]. Recently, some evidence has emerged supporting the use of mHealth for home-based SLT across various patient groups with speech and language disorders including children with articulation disorders, poststroke aphasia, presbyphonia, and voice disorders associated with PD [15-19].

mHealth apps designed for patients with PD have mostly focused on symptom monitoring and providing information or education [20]. Research in this area has mainly addressed specific aspects of care, such as symptom management, medication adherence, physical activity, and exercise interventions [20-22].

However, very few studies have explored the use of mHealth apps for delivering speech therapy to patients with PD. Maas et al [23] proposed a study protocol for a single-blind randomized controlled trial using the Voice Trainer app and an online platform that provides real-time feedback, enabling patients to practice at home alongside remote therapy. The study aimed to evaluate the effectiveness of personalized, home-based speech therapy on quality of life, speech intelligibility, and social participation in individuals with PD who have reduced speech intelligibility. However, no follow-up research has been published to date. Lo et al [18] developed a speech rehabilitation app specifically for patients with PD and validated its usability with 2 participants, reporting improvements in articulation and volume after short-term use. Horin et al [19] investigated the utility of a mHealth app designed for the comprehensive treatment of PD symptoms, including gait, dexterity, and speech, and concluded that the application alone was insufficient to effectively address gait, speech, or dexterity symptoms in PD.

While the use of mHealth apps for speech therapy in patients with PD is still in its early stages, there is potential for significant advancement through ongoing research and technological development. mHealth offers great benefits in terms of accessibility and cost, especially for patients with PD who gradually experience reduced mobility, as it allows them to access therapy from anywhere with a mobile device [24-26]. Furthermore, it provides patients with the opportunity to practice independently without time constraints, and it can automatically measure, collect, and store data, thereby reducing the workload for clinicians.

This study aims to evaluate the feasibility of using a smartphone app to provide self-delivered voice therapy for patients with PD with minimal involvement from SLPs. It also seeks to explore patient satisfaction with the app and its potential effectiveness as a therapeutic tool.

## Methods

### Participants and Recruitment Procedures

Participants were recruited from the Movement Disorders Clinic of the Department of Neurology at Seoul National University Hospital, Seoul, South Korea from September to November 2023. Recruitment was conducted through clinical referrals by neurologists during routine outpatient visits. When neurologists identified patients who presented with speech or voice problems, they referred them to the SLPs for further evaluation. The referrals were based on the neurologists’ clinical judgment at the clinic. Once referred, SLPs independently conducted auditory-perceptual evaluations to confirm the presence of speech and voice issues. The inclusion criteria which were (1) patients with PD reporting voice problems and speech difficulties with these issues confirmed by both SLPs through auditory-perceptual evaluation; each SLP having over 5 years of clinical experience, independently verified the presence of voice problems in all patients; (2) Android smartphone users without visual or auditory difficulties in using apps; and (3) not having laryngeal dysfunctions caused by other diseases. Exclusion criteria which were (1) being illiterate, (2) having a recent (<6 months) history of speech therapy, (3) difficulty in operating a smartphone due to cognitive and motor dysfunction, and (4) having limited access to the internet at home.

### Study Design

This study is a single-arm, rater-blinded, and pretest and posttest design to evaluate the feasibility and, satisfaction of an mHealth program among individuals with PD. The preliminary effectiveness of the app was also assessed.

### mHealth App and Training Program

**Figure 1:**
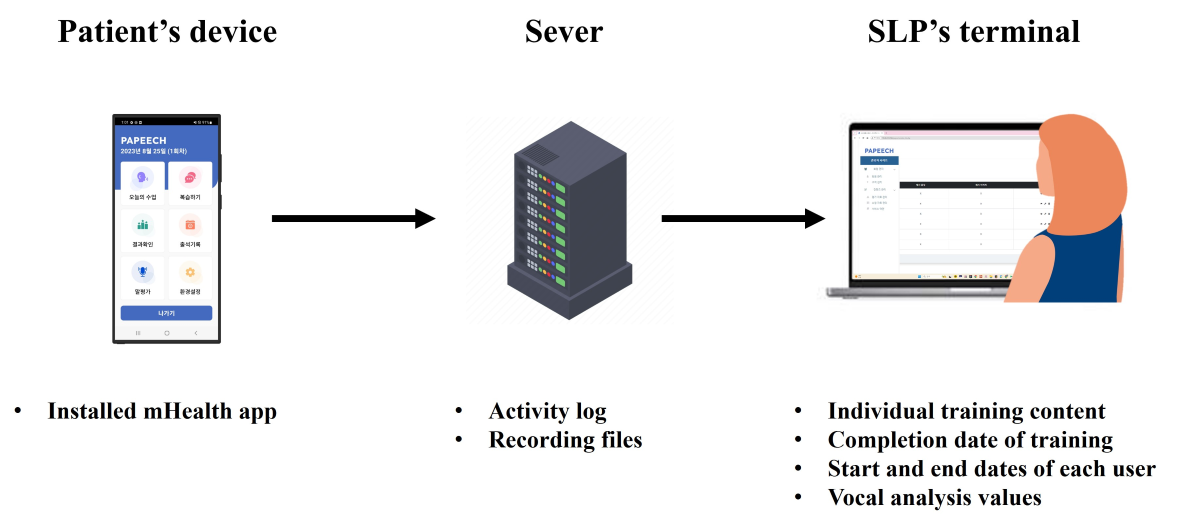
A simplified overview of the composition of the mobile health (mHealth) app used for voice and speech training in Parkinson disease patients. The app comprises 3 main components. SLP: speech-language pathologist.

We developed an Android-based smartphone app for voice and speech training (Figure 1). The app consists of a patient’s device (patient’s own smartphone), a server, and a SLP’s terminal (laptop or desktop computer). All the content for patient evaluation and training, which was developed by the SLPs, is stored on the server.

Participants downloaded the mHealth app onto their smartphones at the hospital. Afterward, they were invited to complete a 5-week home-based training program following the registration and user approval process for participation. The SLPs ensured that the installed app was functioning correctly and educated the participants on how to use it. The server transmitted the content to the devices of approved participants. This content was designed with a user-friendly interface and experience. A sufficiently large font size was used to accommodate older users, and the size and spacing of the buttons were appropriately adjusted to consider motor symptoms such as hand tremors. In addition, if the participants have used a smartphone before, the design was made simple and intuitive enough for them to operate without difficulty. This is because the goal was for patients to use the app independently for evaluation and training during the study, without assistance from clinicians, including SLPs, or caregivers.

All speeches practiced in the session were recorded. The recorded files and the associated activity logs are all stored on the server. The SLP’s terminal is designed for monitoring the entire process, allowing the SLP to check each participant’s start and end dates of training, their individual training content, and the analysis of the recorded speeches within the training. SLPs did not interact with the patient or server in real time during evaluation or training.

**Figure 2:**
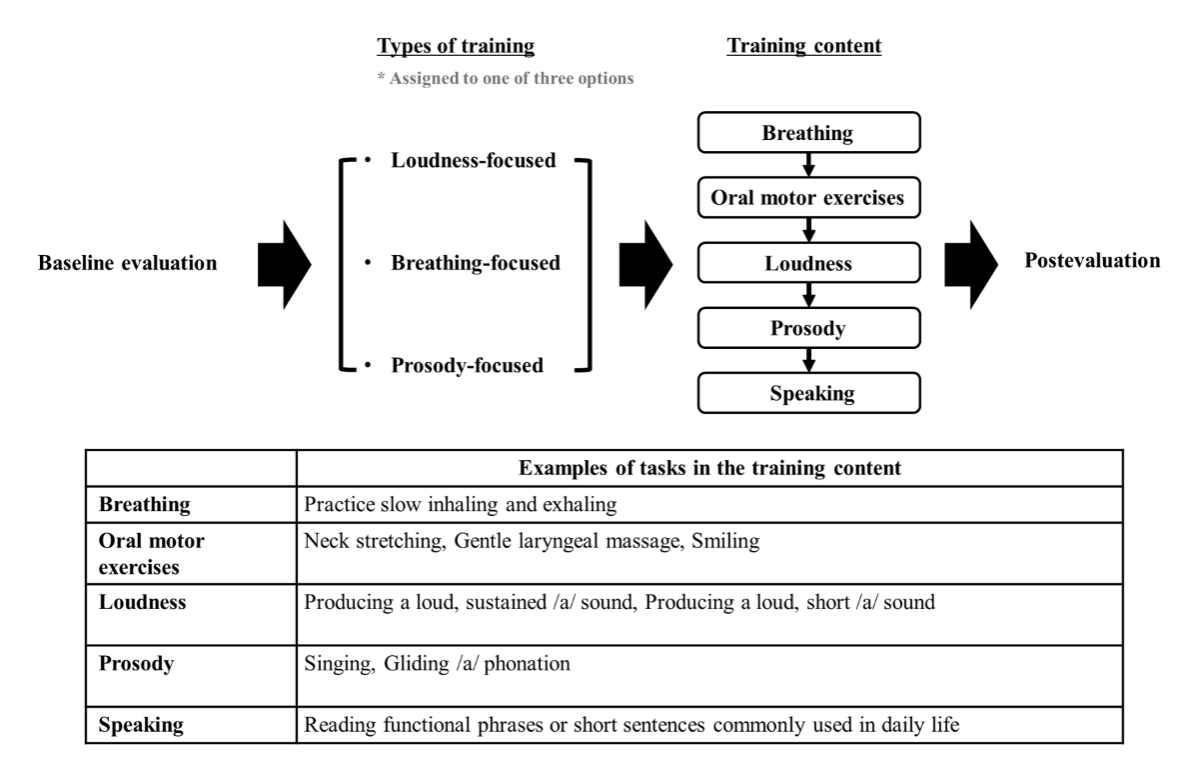
Contents and structure of the 5-week speech training program for participants with Parkinson disease patients using the mHealth app.

Based on the results of the self-administered baseline evaluation (refer below), each participant was assigned to one of 3 different types of training: loudness-focused, breathing-focused, and prosody-focused training. Each training type is designed to include a variety of tasks that target the specific focused area more effectively. The training content comprised 5 stages: breathing, oral motor exercises, loudness, prosody, and speaking (Figure 2). The breathing stage addresses the energy source for speech. PD patients often experience weak or shallow breathing, which can reduce speech volume and stamina. Strengthening respiratory muscles improves airflow, enabling clearer and more sustained speech [27]. Oral motor exercises target the strength and agility of oral muscles, which are often reduced in PD, leading to unclear speech and difficulty swallowing. Strengthening these muscles enhances articulation and supports swallowing function [28]. Loudness training focuses on hypophonia, or reduced speech volume, which is common in PD. Loudness exercises retrain patients to use stronger vocal effort, helping them communicate more effectively in daily conversations [29]. Prosody training helps address the monotone voice often seen in PD, which can make speech sound flat and less engaging. Prosody exercises enhance speech expressiveness, allowing patients to convey meaning more effectively [30].

Finally, functional speaking practice supports patients in initiating and maintaining fluid conversations. This stage builds confidence, improves speech flow, and integrates the skills learned in breathing, loudness, and prosody exercises [31].

This 5-week home-based training program consists of a total of 20 sessions, each lasting approximately 20 minutes to 30 minutes. Participants were instructed to use the app at least 4 days a week, one session a day. To minimize attrition, SLPs monitored the participant’s daily use of the app using the dashboard equipped in the SLPs’ terminal. If necessary, SLPs called the patients to encourage training. The app also includes a pop-up alarm to remind users of each day’s training.

### Measuring Adherence, Satisfaction, and Effectiveness

To verify adherence, we created a webpage for SLPs to monitor the participants’ completion of app training sessions in real-time. The overall adherence was calculated by dividing the number of training sessions completed by the total number of sessions prescribed [8]. The number of participants who withdrew from the study was also identified.

The Satisfaction survey was conducted by phone within a week of either completing or discontinuing the app’s use. Their satisfaction levels were gauged using a 5-point Likert scale anchored by “very satisfied” (5) to “very dissatisfied” (1), aiming to derive the Customer Satisfaction (CSAT) score. The CSAT score is calculated by dividing the number of positive responses (those who are rated 4 or 5) by the total number of responses.

To assess the effectiveness, we conducted both acoustic analysis and auditory perceptual assessments at baseline and at the completion of app use. The tasks included sustained phonation of the vowel /a/, reading 3 short sentences, and reading a 28-word passage from a Korean standardized “Ga-eul” passage. All tasks were recorded using the built-in microphone of each participant's smartphone and were stored on the server. During the recording, participants were instructed to sit upright and remain still, maintaining a distance of approximately 30 cm between their mouths and the smartphone microphone.

The acoustic analysis was conducted using Praat (University of Amsterdam) software (version 6.4.04) embedded in the app. The analysis included the maximum phonation time of the vowel /a/ and the mean vocal intensity (including pauses) for each task, measured in sound pressure level decibel.

The auditory perceptual assessment was performed on all tasks using the GRBAS scale, which is widely used to assess the severity of voice quality. It contains grade (G), which is the overall degree of hoarseness, roughness (R), breathiness (B), asthenia (A), and strain (S). Out of 2 experienced SLPs, each with over 10 years of clinical experience, conducted the ratings on a 4-point Likert scale from 0 (normal) to 3 (severe). The baseline and posttraining evaluation voice files pair were provided in random order in WAV (Waveform audio format) without any identifying information about the order. Furthermore, participants who completed all app usage were asked, during a phone survey, to self-report any changes in their overall voice quality after the training.

### Statistical Analysis

Data were analyzed using SPSS (version 29.0; IBM Corp) for Windows. Frequency analysis was conducted to examine adherence, satisfaction, and self-reported voice quality changes. Paired sample *t* tests were conducted to determine if there were significant changes in the maximum phonation time during sustained phonation of the vowel /a/ before and after app usage, as well as to assess for significant changes in vocal intensity in all tasks. These tests were chosen to compare pre- and postintervention measures for the same participants. The normality of the data was tested using the Shapiro-Wilk test. The results indicated that intensity scores were normally distributed with a *P* value of *P*=.34. Based on this, we chose parametric tests for subsequent analysis. To analyze the reliability of the average agreement rate between evaluators for the auditory perceptual assessment of GRBAS scores, intraclass correlation coefficients (ICCs) were calculated.

### Ethical Considerations

This study was approved by the Seoul National University Hospital Ethics Committee (H-2308-040-1456 and H-2401-010-1499). The research adhered to the principles outlined in the Declaration of Helsinki, and all procedures were conducted in compliance with ethical standards for research involving human participants. All participants provided informed written consent before their participation. They were informed about the study’s purpose, procedures, voluntary nature, and their right to withdraw at any time without consequences. The consent process ensured participants understood that their data would be used for research purposes and protected under the approved ethical guidelines. Data were securely stored on an encrypted server accessible only to authorized research personnel. Participants were not monetarily compensated for their participation.

## Results

### Participants’ Characteristics and Adherence

Thirty patients with PD were initially recruited, but 2 withdrew due to health problems not related to PD. A total of 28 patients with PD participated in the study and used the app. Participant characteristics are summarized in Table 1. Details regarding participant recruitment are provided in Multimedia Appendix 1. Based on the results of baseline evaluation, 20 were assigned to the loudness-focused training program and the remaining 8 participated in the breathing-focused training program. None were assigned to the prosody-focused training program. Out of 25 participants (15 male, 10 female; mean age 68.04, SD 7.81 years; mean disease duration 7.32, SD 4.78 years; H and Y scale ranged from 1 to 4, mean 2.39, SD 0.88) completed all the training sessions while 3 dropped out. One participant could not be reached and the other 2 dropped out due to (1) problems with the internet connection and (2) being too busy, respectively. They completed only 12, 3, and 4 sessions respectively.

**Figure 3:**
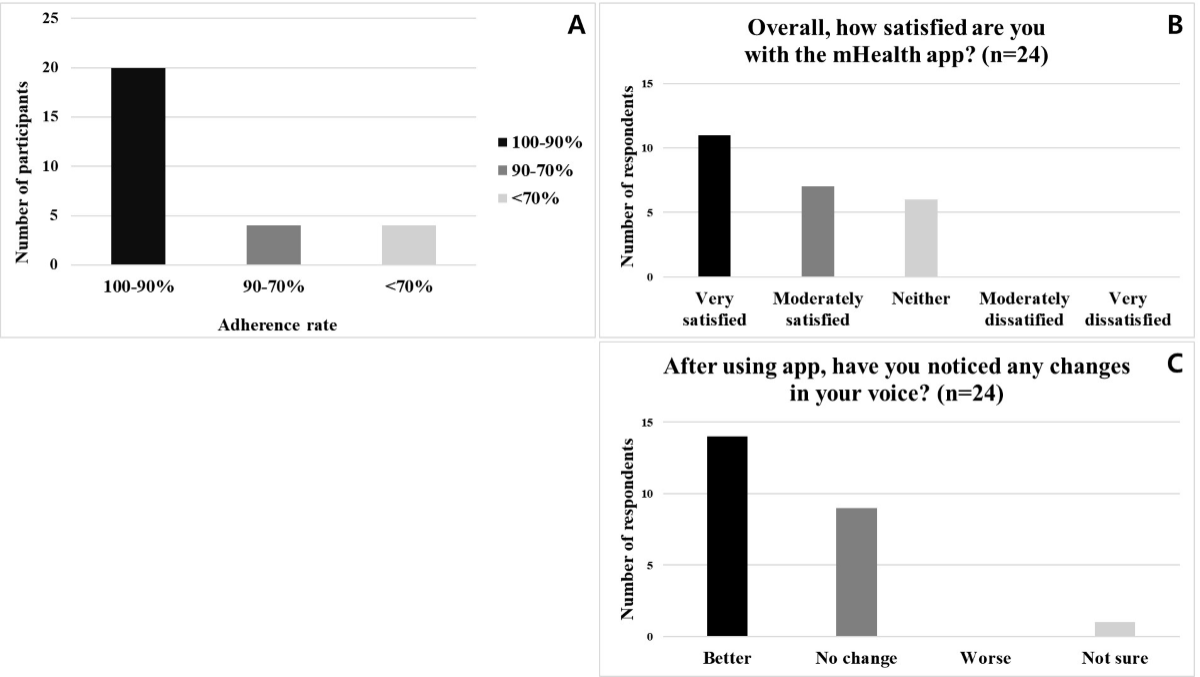
Adherence, satisfaction, and self-reported voice changes among participants using the mobile health (mHealth) app for voice and speech training. (A) Adherence: The percentage of participants who completed the 5-week training program (20 sessions) using the mHealth app. (B) Participant satisfaction: Using a 5-point Likert scale. (C) The self-report of changing voice: Participants’ subjective assessments of whether their voice improved, stayed the same, worsened, or if they were not sure after completing the training program.

The adherence was above 90% of 20 participants, 70-90% in 4, and below 70% in 4 (Figure 3A).

**Table 1:** Demographic and clinical characteristics of the participants with Parkinson disease (N=28), recruited from the Movement Disorders Clinic, Seoul National University Hospital, Seoul, South Korea, between September and November 2023.

Subgroup		Value
Age (years), mean (SD; range)		67.5 (8.16; 51-81)
**Sex, n (%)**		
	Male	17 (60.7)
	Female	11 (39.3)
Disease duration (years), mean (SD; range)		7.54 (4.73; 1-19)
**H and Y scale score (mean 2.39, SD 0.88, median 2, IQR 2–3), n (%)**		
	1	3 (10.7)
	2	15 (53.6)
	3	6 (21.4)
	4	4 (14.3)
**Type of training program, n (%)**		
	Loudness-focused	20 (71.4)
	Breathing-focused	8 (28.6)
	Prosody-focused	0 (0)

### Satisfaction

After using the app, 24 participants took part in the survey on satisfaction (Figure 3B). Out of 11 (45.83%) responded “very satisfied,” 7 (29.17%) “moderately satisfied,” 6 (25%) “neither” and none answered, “moderately dissatisfied” or “very dissatisfied.” The CSAT score was 75%. Most were satisfied with the convenience of being able to practice at their own home, citing it as the primary reason. None reported any technical difficulties.

### Effectiveness

Acoustic analysis and perceptual assessment results are presented in Table 2. Significant differences were observed in all acoustic measurements (*P*<.05). Significant differences in voice quality in all components of the GRBAS scale were observed before and after using the app. The inter-rater reliability between the 2 raters was fair too good (ICC=0.440). Among the 24 who took part in the survey (Figure 3C), 14 (58.33%) reported their voice “getting better,” 9 (37.50%) reported “no change,” 1 (4.17%) reported being “unsure,” and none reported “getting worse.”

**Table 2:** Pre- and postintervention changes in maximum phonation time (MPT), vocal intensity, and GRBAS^a^ scale scores in participants with Parkinson disease (N=25).

Variable			Before		After		*t* test (*df*) or *z* score			*P* value
MPT (seconds)			11.15 (5.38)		14.01 (5.64)		–3.273 (24)		.003	
**Vocal intensity, mean (SD)**										
	Sustained phonation (dB)	72.32 (5.04)		75.67 (3.75)		–4.761 (24)		<.001		
	Reading short sentences (dB)	72.59 (4.09)		74.04 (4.08)		–3.164 (24)		.004		
	Reading “Ga-eul” passage (dB)	69.64 (4.55)		71.59 (3.61)		–3.546 (23)		.002		
	All tasks (dB)	71.59 (4.39)		73.81 (3.48)		–4.462 (24)		<.001		
**Variable (GRBAS** **scale score)**										
	Grade	1 (2)		1 (1)		–4.601		<.001		
	Roughness	0 (2)		0 (1)		–3.804		<.001		
	Breathiness	1 (2)		0 (1)		–5.959		<.001		
	Asthenia	0 (1)		0 (0)		–6.130		<.001		
	Strain	0 (1)		0 (1)		–3.930		<.001		

^a^GRBAS: grade, roughness, breathiness, asthenia, and strain.

## Discussion

### Principal Findings

The aim of this study was to investigate the feasibility of patients with PD independently performing voice and speech training at home using a smartphone app as an alternative to traditional face-to-face therapy. The study involved SLPs in developing a mHealth app based on speech training content commonly used on patients with PD, and evaluated patients’ adherence to treatment schedules, satisfaction with app-based therapy, and treatment effectiveness.

Our study indicates that speech therapy delivered through an mHealth app is a feasible solution for patients with PD. A total of 20 out of 25 participants who completed the app showed an adherence rate of over 90%. Given that dropout rates in fields requiring various health behavior changes reach 30%-60%, an adherence rate of over 90% can be considered sufficiently high and indicative of successful adherence [32]. It is typically stated that an adherence rate of over 80% is necessary for optimal therapeutic efficacy, and our study has achieved this.

About 75% of participants were satisfied with the app use and improvements in voice and speech were observed both in objective and subjective evaluations. This is similar to the satisfaction rate (70%) of an internet-based telerehabilitation application for speech therapy [33] and is also comparable to a study reporting satisfaction rate of 82.6% with the smartphone app for people with aphasia [34].

Another notable point is that positive results were obtained for both objective and subjective ratings for voice. It is generally known that acoustic assessments highly correlate with SLP’s auditory perceptual ratings but have a low correlation with patient subjective assessments [35,36]. This indicates that there is a discrepancy between the degree of voice impairment assessed by the assessor and by the patients. The consistency in our results among 3 methods of evaluation indicates the robustness of effectiveness.

The advancement of mobile and electronic technologies has increased interest in non–face-to-face therapy, which has been further accelerated by the COVID-19 pandemic. This can help address medical accessibility issues. If non–face-to-face methods are proven to be as usable and effective as face-to-face therapy, they will be a more convenient way to receive medical services. Previous studies have confirmed its feasibility and effectiveness in various rehabilitation fields, such as speech, motor, and cognition [10,37,38]. In addition, the benefits of online treatment reported include time and cost savings and reduced caregiver burden. Along with these benefits, a key advantage of mHealth apps is that they enable patients to take an active role in managing their condition. Previous studies indicate that the integration of mHealth technology into therapy is an optimized clinical pathway for patients [6]. Although the number of studies is limited compared to real-time teletherapy provided by therapists, there are a few that have confirmed the usefulness and effectiveness of app-based therapies [15,17,18,39]. These include reports of significant improvements in pronunciation and speech volume in people with PD who received speech therapy using homegrown software [18]. This finding is in line with our results and illustrates the potential of the mHealth app as a feasible approach to speech rehabilitation.

In contrast to the high adherence in our study, a previous study investigating the usability of an mHealth app developed to address walking, speech, and dexterity symptoms in patients with PD reported low adherence and effectiveness [19]. The adherence to speech exercises was 66.8% (SD 26.5%). In the study, participants in the intervention group were instructed to independently complete exercises using the provided mHealth app on their personal smartphones once a day for approximately 90 days. This app required practicing various areas such as mobility exercises in addition to speech, and it took about 30 minutes to complete all sessions. This may have placed a physical and mental burden on the users.

In addition, considering that appropriate feedback and interaction with therapists generally impact the adherence and effectiveness of therapy, the extent to which sufficient feedback was automatically provided within the app may have also influenced these outcomes. In our app, not only does it provide reminder functions through pop-up alarms, but it also provides different feedback each time whenever the patient performs a task (eg, “Good job! Squeeze your abdomen and speak louder!”). Such feedback may have positively influenced the patient’s continued use. Furthermore, over 50% of the participants in the previous study experienced technological issues with the app, which may have interfered with the outcomes. In our app, we tried to create a user interface older adults friendly as much as possible and to ensure the seamless operation of the app. As a result, there were no reports of technical issues, and this also could have affected adherence, effectiveness, and satisfaction.

### Limitations and Future Directions

However, our study has several limitations. Because the primary aim of this study was to evaluate the feasibility and it was an uncontrolled study, the effectiveness observed in our study should be interpreted with caution. Larger randomized controlled trials are needed to provide more robust results on effectiveness. In addition, consideration of factors that may affect the usability of the mHealth app, such as disease severity and digital literacy [40] of patients from the recruitment stage, would be useful to evaluate the potential for introducing this app to a broader population of individuals with PD.

Second, we were unable to conduct functional assessments to determine whether participants’ voice issues improved in real-life communication situations. While the Voice-Related Quality of Life tool does not directly measure improvements in voice use during real-life situations, it provides valuable insights into how patients perceive the impact of their voice disorder on their quality of life. Studies have demonstrated that after face-to-face speech therapy, such as the Lee Silverman Voice Treatment or the Speak Out Voice program, patients exhibit improved Voice-Related Quality of Life scores [41,42].

Using the Dysarthria Impact Questionnaire to evaluate the psychosocial effects of dysarthria could also provide meaningful insights [43]. In addition, assessing effectiveness using data collected from naturalistic environments, such as real-life conversational settings, could yield more reliable results. Including evaluations from primary caregivers to determine whether patients’ speech problems have improved would provide a more objective perspective. Patients with PD often face challenges with self-monitoring due to the nature of the disease, making patient-reported improvements insufficient [44]. Therefore, evaluations should integrate diverse perspectives and methodologies.

Third, our study encountered difficulties in achieving consistent calibration across all participants’ devices. The variations in smartphone models and built-in microphone sensitivities presented challenges in standardizing intensity calibration. According to Park and Lee [45], differences in microphone characteristics among smartphone models can lead to discrepancies in recording and analysis, with omnidirectional microphones, in particular, being more susceptible to ambient noise. They suggested that using a unidirectional wired lapel microphone could offer more consistent recording quality. Consequently, we recommended that participants maintain a specific distance from the microphone and minimize background noise, providing tutorial videos within the app to facilitate consistent recording practices. However, hardware differences among devices may still impact the consistency of intensity measurements. This suggests that future research may benefit from implementing additional calibration methods to improve measurement reliability.

Fourth, while this app was designed as a tool to substitute face-to-face therapy, it currently faces limitations due to its inability to fully replicate the real-time feedback and personalized adjustments provided during in-person therapy. To overcome these challenges, future research should focus on improving the app’s functionality and design.

Specifically, integrating customized feedback mechanisms and improved monitoring systems is crucial. Without these features, participants may risk developing vocal abuse by overcompensating with improper techniques. Continuous monitoring can help identify and resolve any discomfort or tension early, ensuring the app’s safety and effectiveness. These improvements could help bridge the gap between app-based therapy and face-to-face therapy. In this process, it is also important to investigate the app’s impact on the overall quality of life of patients with PD compared to traditional in-person therapy. Widely used tools such as the World Health Organization Quality of Life Questionnaire or Parkinson Disease Questionnaire-39 can be used for this purpose [46].

In addition, understanding the impact of self-managed mHealth apps on caregiver burden, particularly from the perspective of primary caregivers, is an important research topic. If such apps enable patients to manage themselves more effectively, they could reduce the caregiving burden, alleviating stress and fatigue for caregivers. This would not only enhance the quality of life for caregivers but could also have a positive impact on the patient-caregiver relationship. Therefore, such analyses are crucial for evaluating whether the app provides meaningful benefits for both patients and their caregivers.

Finally, future research could consider longitudinal studies to evaluate the long-term effects and sustainability of mHealth apps. Patient adherence is crucial for treatment effectiveness and improvement in their health status. Nevertheless, most existing studies have short durations, limiting research on factors affecting mHealth app adherence [47]. Tracking changes in user adherence over extended periods could help develop user retention strategies. Furthermore, the accumulated data could be used to analyze user behavior patterns and develop predictive models.

### Conclusions

Despite the small sample size of this study, this study supports the feasibility of self-delivered speech therapy at home for patients with PD. Especially considering the age of the participants, the mHealth app we developed is not only suitable for older users but also proves to be satisfying for them.

## Appendix

Multimedia Appendix 1 Details regarding participant recruitment.

## Abbreviations

CSAT: Customer Satisfaction
GRBAS: grade, roughness, breathiness, asthenia, and strain
ICC: intraclass correlation coefficient
mHealth: mobile health
PD: Parkinson disease
SLP: speech-language pathologist
SLT: speech-language therapy
WAV: Waveform audio format

## Data Availability

The datasets generated during this study are not publicly available due to the small sample size, which requires protecting the privacy of participants, but are available from the corresponding author on reasonable request.

## References

[1] Logemann JA, Fisher HB, Boshes B, Blonsky ER (1978). Frequency and cooccurrence of vocal tract dysfunctions in the speech of a large sample of parkinson patients. J Speech Hear Disord.

[2] van Hooren MRA, Baijens LWJ, Vos R, Pilz W, Kuijpers LMF, Kremer B, Michou E (2016). Voice- and swallow-related quality of life in idiopathic parkinson's disease. Laryngoscope.

[3] Gillivan-Murphy P, Miller N, Carding P (2019). Voice treatment in Parkinson’s disease: patient perspectives. JPRLS.

[4] Jones CH, Dolsten M (2024). Healthcare on the brink: navigating the challenges of an aging society in the United States. NPJ Aging.

[5] Weidner K, Lowman J (2020). Telepractice for adult speech-language pathology services: a systematic review. Perspect ASHA SIGs.

[6] Theodoros D, Hill A, Russell T (2016). Clinical and quality of life outcomes of speech treatment for parkinson's disease delivered to the home via telerehabilitation: a noninferiority randomized controlled trial. Am J Speech Lang Pathol.

[7] Griffin M, Bentley J, Shanks J, Wood C (2018). The effectiveness of lee silverman voice treatment therapy issued interactively through an iPad device: a non-inferiority study. J Telemed Telecare.

[8] Quinn R, Park S, Theodoros D, Hill AJ (2019). Delivering group speech maintenance therapy via telerehabilitation to people with parkinson's disease: a pilot study. Int J Speech Lang Pathol.

[9] Dias AE, Limongi JCP, Barbosa ER, Hsing WT (2016). Voice telerehabilitation in parkinson's disease. Codas.

[10] Chang HJ, Kim J, Joo JY, Kim H (2023). Feasibility and efficacy of video-call speech therapy in patients with parkinson's disease: a preliminary study. Parkinsonism Relat Disord.

[11] World Health Organization (2011). mHealth: new horizons for health through mobile technologies: second global survey on eHealth.

[12] Gorski I, Bram JT, Sutermaster S, Eckman M, Mehta K (2016). Value propositions of mHealth projects. J Med Eng Technol.

[13] Rowland SP, Fitzgerald JE, Holme T, Powell J, McGregor A (2020). What is the clinical value of mHealth for patients?. NPJ Digit Med.

[14] Iribarren SJ, Akande TO, Kamp KJ, Barry D, Kader YG, Suelzer E (2021). Effectiveness of mobile apps to promote health and manage disease: systematic review and meta-analysis of randomized controlled trials. JMIR Mhealth Uhealth.

[15] Kim SY, Song M, Jo Y, Jung Y, You H, Ko MH, Kim GW (2023). Effect of voice and articulation parameters of a home-based serious game for speech therapy in children with articulation disorder: prospective single-arm clinical trial. JMIR Serious Games.

[16] Liu H, Cordella C, Ishwar P, Betke M, Kiran S (2023). Consistent long-term practice leads to consistent improvement: benefits of self-managed therapy for language and cognitive deficits using a digital therapeutic. Front Digit Health.

[17] Cho NB, Cho SR, Choi SH, You H, Nam SI, Kim H (2021). Short-term and long-term efficacy of oropharyngolaryngeal strengthening training on voice using a mobile healthcare application in elderly women. Commun Sci Disord.

[18] Lo HC, Tang ST, Wei WL, Chuang CC (2024). Design and usability evaluation of speech rehabilitation app interface for patients with Parkinson's disease.

[19] Horin AP, McNeely ME, Harrison EC, Myers PS, Sutter EN, Rawson KS, Earhart GM (2019). Usability of a daily mHealth application designed to address mobility, speech and dexterity in parkinson's disease. Neurodegener Dis Manag.

[20] Estévez S, Cambronero M, García-Ruiz Y, Llana Díaz LF (2019). Mobile applications for people with parkinson's disease: a systematic search in app stores and content review. J Univers Comput Sci.

[21] Triantafyllidis A, Segkouli S, Zygouris S, Michailidou C, Avgerinakis K, Fappa E, Vassiliades S, Bougea A, Papagiannakis N, Katakis I, Mathioudis E, Sorici A, Bajenaru L, Tageo V, Camonita F, Magga-Nteve C, Vrochidis S, Pedullà L, Brichetto G, Tsakanikas P, Votis K, Tzovaras D (2023). Mobile app interventions for parkinson's disease, multiple sclerosis and stroke: a systematic literature review. Sensors (Basel).

[22] Kim A, Yun SJ, Sung KS, Kim Y, Jo JY, Cho H, Park K, Oh BM, Seo HG (2021). Exercise management using a mobile app in patients with parkinsonism: prospective, open-label, single-arm pilot study. JMIR Mhealth Uhealth.

[23] Maas JJL, De Vries NM, Bloem BR, Kalf JG (2022). Design of the PERSPECTIVE study: PERsonalized SPEeCh therapy for actIVE conversation in parkinson's disease (randomized controlled trial). Trials.

[24] Theodoros D, Aldridge D, Hill A, Russell T (2019). Technology-enabled management of communication and swallowing disorders in parkinson's disease: a systematic scoping review. Int J Lang Commun Disord.

[25] Theodoros D (2021). Telerehabilitation for communication and swallowing disorders in parkinson's disease. J Parkinsons Dis.

[26] Vaezipour A, Campbell J, Theodoros D, Russell T (2020). Mobile apps for speech-language therapy in adults with communication disorders: review of content and quality. JMIR Mhealth Uhealth.

[27] Zhuang J, Jia J (2022). Effects of respiratory muscle strength training on respiratory-related impairments of parkinson's disease. Front Aging Neurosci.

[28] Wang CM, Shieh WY, Ho CS, Hu YW, Wu YR (2018). Home-based orolingual exercise improves the coordination of swallowing and respiration in early parkinson disease: a quasi-experimental before-and-after exercise program study. Front Neurol.

[29] Ramig LO, Countryman S, Thompson LL, Horii Y (1995). Comparison of two forms of intensive speech treatment for parkinson disease. J Speech Hear Res.

[30] Stegemöller EL, Radig H, Hibbing P, Wingate J, Sapienza C (2017). Effects of singing on voice, respiratory control and quality of life in persons with parkinson's disease. Disabil Rehabil.

[31] Barnish MS, Horton SMC, Butterfint ZR, Clark AB, Atkinson RA, Deane KHO (2017). Speech and communication in parkinson's disease: a cross-sectional exploratory study in the UK. BMJ Open.

[32] Litts JK, Abaza MM (2017). Does a multidisciplinary approach to voice and swallowing disorders improve therapy adherence and outcomes?. Laryngoscope.

[33] Theodoros DG, Constantinescu G, Russell TG, Ward EC, Wilson SJ, Wootton R (2006). Treating the speech disorder in parkinson's disease online. J Telemed Telecare.

[34] Choi YH, Park HK, Paik NJ (2016). A telerehabilitation approach for chronic aphasia following stroke. Telemed J E Health.

[35] Karnell MP, Melton SD, Childes JM, Coleman TC, Dailey SA, Hoffman HT (2007). Reliability of clinician-based (GRBAS and CAPE-V) and patient-based (V-RQOL and IPVI) documentation of voice disorders. J Voice.

[36] Woisard V, Bodin S, Yardeni E, Puech M (2007). The voice handicap index: correlation between subjective patient response and quantitative assessment of voice. J Voice.

[37] Federico S, Cacciante L, Cieślik B, Turolla A, Agostini M, Kiper P, Picelli A, RIN_TR_Group (2024). Telerehabilitation for neurological motor impairment: a systematic review and meta-analysis on quality of life, satisfaction, and acceptance in stroke, multiple sclerosis, and parkinson's disease. J Clin Med.

[38] Schoenberg MR, Ruwe WD, Dawson K, McDonald NB, Houston B, Forducey PG (2008). Comparison of functional outcomes and treatment cost between a computer-based cognitive rehabilitation teletherapy program and a face-to-face rehabilitation program. Professional Psychology: Research and Practice.

[39] Hutchison MG, Di Battista AP, Loenhart MM (2023). A continuous aerobic resistance exercise protocol for concussion rehabilitation delivered remotely via a mobile app: feasibility study. JMIR Form Res.

[40] Esper CD, Valdovinos BY, Schneider RB (2024). The importance of digital health literacy in an evolving parkinson's disease care system. J Parkinsons Dis.

[41] Behrman A, Cody J, Elandary S, Flom P, Chitnis S (2020). The effect of SPEAK OUT! and the LOUD crowd on dysarthria due to parkinson's disease. Am J Speech Lang Pathol.

[42] Parveen S (2020). Group-based intervention of participants with parkinson disease: findings from a 6-month LOUD Crowd® program. Clin Arch Commun Disord.

[43] Walshe M, Peach R, Miller N (2009). Dysarthria impact profile: development of a scale to measure psychosocial effects. Int J Lang Commun Disord.

[44] Contreras-Ruston F, Castillo-Allendes A, Saavedra-Garrido J, Ochoa-Muñoz AF, Hunter EJ, Kotz SA, Navarra J (2024). Voice self-assessment in individuals with parkinson's disease as compared to general voice disorders. Parkinsonism Relat Disord.

[45] Park JI, Lee SJ (2024). A comparison of acoustic measures among the microphone types for smartphone recordings in normal adults. Phonet Speech Sci.

[46] Zhao N, Yang Y, Zhang L, Zhang Q, Balbuena L, Ungvari GS, Zang YF, Xiang YT (2021). Quality of life in parkinson's disease: a systematic review and meta-analysis of comparative studies. CNS Neurosci Ther.

[47] Jakob R, Harperink S, Rudolf AM, Fleisch E, Haug S, Mair JL, Salamanca-Sanabria A, Kowatsch T (2022). Factors influencing adherence to mHealth apps for prevention or management of noncommunicable diseases: systematic review. J Med Internet Res.

