# Supplementary figures and images for: Smartphone Application–Based Voice and Speech Training Program for Parkinson Disease: Feasibility and Satisfaction Study With a Preliminary Rater-Blinded Single-Arm Pretest and Posttest Design
Source: J Med Internet Res. 2025 Feb 13;27:e63166. doi: 10.2196/63166 (PMC11888063; doi:10.2196/63166)

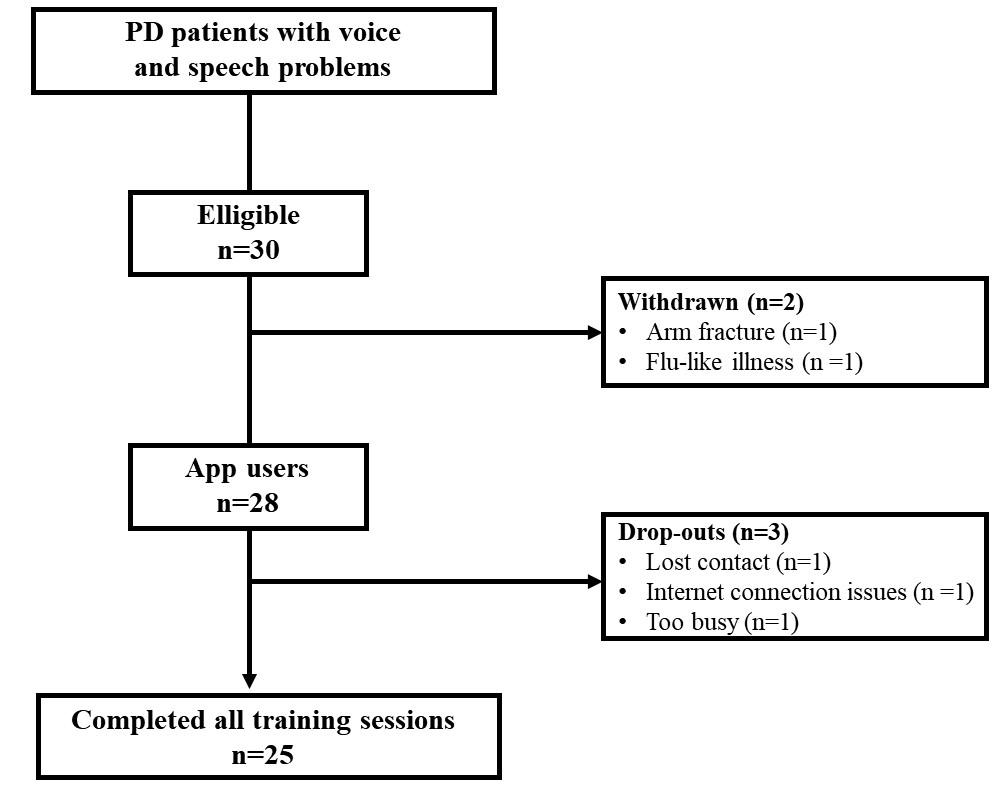

Supplement: Multimedia Appendix 1 [file jmir_v27i1e63166_app1.png]
